# Environmental Exposures and ADHD

**DOI:** 10.1289/ehp.10274

**Published:** 2007-08

**Authors:** Jack Brondum

**Affiliations:** Hennepin County Department of Human Services and Public Health, Environmental Health and Epidemiology, Hopkins, Minnesota, E-mail: jack.brondum@co.hennepin.mn.us

In their article, “Exposures to Environmental Toxicants and Attention Deficit Hyperactivity Disorder [ADHD] in U.S. Children,” Braun et al. (2006) advanced our knowledge of the effects of environmental tobacco smoke (ETS) and lead on the central nervous system of children. With respect to lead exposure, the study, importantly, focused on an older age group (4–15 years) than is generally studied (< 6 years) because of the greater sensitivity of the developing central nervous system to environmental insult early in life [Centers for Disease Control and Prevention (CDC) 1997].

In the logistic model used by Braun et al. (2006), the association of ADHD with lead exposure was statistically significant in the highest exposure quintile; however, it was also tenuous. Although not unheard of, the cutoff (*p* < 0.2) for inclusion of factors and variables associated with ADHD on univariate analysis was generous compared with the commonly used 0.1 or 0.05, and very close to the *p*-value of the lead–ADHD association of 0.19. The lead–ADHD relationship also exhibited a significant monotonic dose response, so it would have been helpful to know how the authors developed their exposure metric. Why, for example, were quintiles selected rather than another interval scheme, and why were they not of uniform size? Was the reported dose response the only model considered, or did the authors investigate other models, as some have done in studying the relationship of lead exposure and cognition (Canfield et al. 2003)?

Braun et al. (2006) noted that their analyses were limited by the cross-sectional nature of the National Health and Nutrition Examination Survey data they used, precluding adjustment of their model for certain covariates and potential confounders (e.g., parental psychopathology). Based on data from multiple studies, ADHD heritability has been estimated to be about 75% (Biederman and Faraone 2005). Inability to adjust for parental psychopathology is therefore an important limitation, because adjustment would likely reduce—and might eliminate—the associations of ADHD with ETS and lead. In studies of lead exposure and cognition, some of which Braun et al. (2006) cited as being consistent with their findings, the strength of the IQ–lead relationship can be dwarfed by the relationship of IQ to other factors such as parenting and socioeconomic status (Koller et al. 2004). When reporting associations of environmental contaminants and pathology, it seems prudent to maintain a broader perspective, as well as an environmental health perspective.
